# Disease Burden and Cost of Illness of Female Breast Cancer in Poland: A Societal-Perspective Analysis with Cost Attribution to Payers, Patients, and the Economy

**DOI:** 10.3390/healthcare14203434

**Published:** 2026-10-14

**Authors:** Michał Seweryn, Agnieszka Leszczyńska, Małgorzata Budasz-Świderska, Tomasz Banaś, Artur Turek, Paweł Michał Potocki

**Affiliations:** 1 Faculty of Health Sciences, Andrzej Frycz Modrzewski Krakow University, Gustawa Herlinga-Grudzińskiego 1, 30-705 Krakow, Poland; 2 EconMed Europe, Chłopickiego 5, 31-525 Krakow, Poland; 3 Roche Polska, 02-672 Warsaw, Poland; 4 Department of Radiotherapy, Maria Skłodowska-Curie National Research Institute of Oncology, Krakow Branch, 31-115 Krakow, Poland; 5 Chair and Department of Biopharmacy, Faculty of Pharmaceutical Sciences in Sosnowiec, Medical University of Silesia, 40-055 Katowice, Poland; 6 Department of Oncology, Jagiellonian University Medical College, 31-008 Krakow, Poland

**Keywords:** breast cancer, cost-of-illness, disease burden, disability-adjusted life years, DALY, years of life lost, premature mortality, societal perspective, indirect costs, Poland

## Abstract

**Highlights:**

**What are the main findings?**

- Female breast cancer caused 151,355 DALYs in Poland in 2023, with over 90% of the burden due to premature mortality.
- Its core societal cost, based on administrative and registry data, was ≈€1.41 billion, rising to ≈€4.54 billion in an exploratory extended scenario that additionally incorporates patient-reported costs obtained from a convenience sample of 289 women.

**What are the implications of the main findings?**

- Most of the burden falls outside the public payer, on patients, households, and the economy. In the modelled extended scenario, public healthcare expenditure represented approximately 18.5% of the total.
- A societal perspective with indirect costs should inform breast-cancer policy and health technology assessment in Poland.

**Abstract:**

Background/Objectives: No Polish study has combined breast-cancer disease burden with a societal cost of illness attributing costs to their bearers. We estimated disease burden in disability-adjusted life years (DALYs), societal cost of illness, and its distribution across payers, patients, and the economy. Methods: Secondary modelling cost-of-illness analysis of administrative, registry, and public-statistical data (NFZ, ZUS, GUS, NCR) and GBD 2023. Productivity losses used a human-capital approach. Two tiers are reported: a core estimate from administrative and registry sources, and an exploratory extended scenario adding patient-reported components extrapolated nationally from a non-probability sample of 289 women (non-validated questionnaire). Deterministic and probabilistic sensitivity analyses were performed; 95% intervals are model-based simulation intervals. Reference year 2024; mortality 2023. Results: In 2023, breast cancer caused 151,355 DALYs (138,492 YLLs, 12,863 YLDs), 91.5% from premature mortality. The core societal cost was approximately EUR 1.41 billion (EUR 1.34–1.49 billion); NFZ expenditure was EUR 837.7 million. Adding patient-reported costs and time losses, the extended scenario reached EUR 4.54 billion (EUR 3.81–5.40 billion); as its largest components are derived from the convenience sample, it is exploratory, not a national estimate. Indirect costs dominated it; direct medical costs led the core estimate. The public payer bore 18.5% of the extended total, patients/households 41.2%, employers 40.3%. Conclusions: The burden is driven predominantly by premature mortality and productivity loss. Policy inferences should rest on the core estimate; the extended figure is exploratory. Analyses restricted to healthcare budgets capture only part of it, supporting explicit cost attribution alongside the payer perspective.

## 1. Introduction

Cancer is a leading cause of morbidity and mortality worldwide and in Poland, the cancer burden has been rising, placing growing pressure on the health system and on society [1,2,3]. Breast cancer is the most commonly diagnosed malignancy among women; globally, an estimated 2.3 million women were diagnosed and approximately 670,000 died in 2022 [4]. These international figures are provided only as background: the present analysis is focused specifically on Poland, where breast cancer remains a major public-health problem on every level—clinical, economic, and social. It accounts for 25.3% of all malignant neoplasms among women and its incidence continues to rise, according to National Cancer Registry reporting [5], and it generates a substantial and growing economic burden through direct and indirect costs borne by the healthcare payer, patients and their households, employers, and the wider economy [6,7,8].

Direct costs include healthcare and non-healthcare expenditures financed by third-party payers as well as patients’ out-of-pocket spending. Indirect costs are primarily driven by productivity losses due to morbidity, disability, and premature mortality, with measurable consequences for employers and the broader economy. To align with a societal perspective, we attribute costs to specific cost bearers, distinguishing expenditures financed by the healthcare payer and social insurance from out-of-pocket payments by patients/households and productivity losses affecting employers and the wider economy. Three concepts are kept separate throughout. Real resource costs are goods and services actually consumed, such as healthcare services and patient out-of-pocket purchases. Productivity losses are foregone output and are borne by employers and the wider economy. Transfer payments, such as sickness and disability benefits disbursed by the social insurance system, redistribute income rather than consume resources and are therefore not added to the societal total; they indicate who finances a loss, not that an additional loss has occurred. Household income losses are likewise a distributional consequence of the same underlying productivity loss and are not counted separately. Because the diagnostic workup and treatment pathway is often prolonged and multi-stage, breast cancer can also impose considerable financial strain on patients and families. Routine travel to specialised centres for follow-up visits and ongoing therapy requires time and financial resources, and additional out-of-pocket expenses may include supportive medications used during chemotherapy, specialised foods and oral nutritional supplements, and payments for medical services that are not always covered or reimbursed [7,9]. Moreover, the disease may limit or preclude continued employment, reducing household income; in some cases, families must also finance long-term care, further compounding the economic burden and contributing to financial hardship and psychological stress [10].

Despite the growing significance of breast-cancer, its societal impact in Poland has not been comprehensively quantified in a way that simultaneously captures the epidemiological disease burden and the cost of illness across payer expenditures, patient-incurred costs, and productivity losses in both paid and unpaid work. Earlier Polish work has addressed parts of this picture separately: short-term absence costs across neoplasms [2], direct and indirect cancer costs at the population level [3,6], productivity losses and public finance effects of breast cancer for 2010 to 2014 [8], and direct and indirect breast cancer costs for 2017 to 2019 [11]. The systemic report on which part of the present analysis draws [12] presented patient-reported cost categories but neither expressed the burden in DALYs nor attributed costs to the parties bearing them. What has not previously been done for Poland is combining a registry-based DALY estimate with a societal cost of illness in which every component is assigned both to a data tier and to a cost bearer. The primary aim of this study was therefore to estimate the disease burden (in DALYs) and the cost of illness of female breast cancer in Poland from a societal perspective, using administrative, registry, and public-statistical data. A secondary, exploratory aim was to extend the analysis with patient-reported costs—including out-of-pocket expenditures, reduced labour-market participation, presenteeism, unpaid work, and informal caregiving—and to examine how the burden is distributed across the healthcare payer, patients and their households, and the wider economy.

A clearer understanding of the societal burden—and of how costs are distributed across stakeholders—can inform strategies to mitigate financial impacts and support more efficient allocation of resources across treatment, survivorship care (including rehabilitation), and prevention.

## 2. Materials and Methods

### 2.1. Study Design and Analytical Perspective

We conducted a combined disease-burden and cost-of-illness analysis of female breast-cancer in Poland for the reference year 2024. The analysis was restricted to women, who account for approximately 99% of breast cancer cases in Poland, adopting a societal perspective. We quantified (i) the disease burden expressed in DALYs and (ii) direct medical costs, direct non-medical costs, and indirect costs linked to productivity losses among patients and informal caregivers. In addition, we present a complementary patient/household and caregiver perspective, focusing on income losses attributable to sickness absence and reduced working time.

Results are reported in two tiers. The core estimate is based exclusively on administrative and registry sources (NFZ, ZUS, NCR, GUS) and international burden data (IHME/GBD), and comprises public healthcare expenditure, sickness-absence costs, and long-term productivity losses from premature mortality and disability. The extended estimate additionally incorporates patient-reported components—out-of-pocket and non-medical costs, reduced working time, presenteeism, unpaid work, and informal caregiving—obtained as aggregated, previously published figures from a publicly available systemic report on breast cancer in Poland [12]. The core estimate is our primary result; the extended estimate is an exploratory scenario carrying substantially greater uncertainty. It is deliberately not described as an upper bound: because its largest components are extrapolated from a non-probability sample, the direction of any bias cannot be established analytically, and the scenario may either overstate or understate the corresponding national costs.

Each component used the most recent data available at the time of analysis. As national cancer mortality for 2024 had not yet been published, mortality-based estimates (YLLs and premature-mortality productivity loss) used the most recent available NCR data (2023).

Reference periods and harmonisation. The core estimate uses a single monetary reference year, 2024; the extended estimate combines monetary inputs from more than one period, as set out below. NFZ expenditure, ZUS sickness absence, GDP, and employment counts all refer to 2024. Mortality-based components (YLLs and the premature-mortality productivity loss) use National Cancer Registry data for 2023, the most recent year published at the time of analysis, and are valued at 2024 productivity levels. YLDs are GBD 2023 estimates. The patient-reported components originate from a survey fielded between June and September 2025 in which respondents reported expenditures and time losses for the preceding 12 months; the resulting per-patient values were therefore treated as referring to a 12-month treatment year. Two operations should be distinguished here. First, the per-patient values were applied to the 2024 treated population, so that the quantity base of the extended components is 2024. Second, and separately, those values were not re-expressed in 2024 prices: no price indexation was applied between the survey recall period and the monetary reference year. The extended estimate therefore incorporates mixed-period monetary inputs, combining administrative and registry components valued at 2024 prices with patient-reported components that carry the price level of the 2024 to 2025 period in which they were reported. This is a departure from a strictly constant-price analysis, and it is noted among the limitations. All values were calculated in PLN and converted to EUR at a single reference rate.

### 2.2. Data Sources and Study Populations

Cost estimates combined administrative data with previously published, aggregated patient-reported information. Public healthcare expenditures (2024) were obtained from the National Health Fund (NFZ) for services attributed to breast cancer (ICD-10 C50) and oncology treatment episodes coded as Z51 with concurrent C50. Productivity-related parameters (GDP and employment counts, 2024) were obtained from Central Statistical Office (GUS) publications [13,14]. Sickness-absence volumes (2024) were derived from Social Insurance Institution (ZUS) reporting for ICD-10 C50 [15]. Mortality inputs (most recent available year, 2023) were obtained from the National Cancer Registry (NCR) [16], and years lived with disability (YLDs, 2023) were taken from the Institute for Health Metrics and Evaluation (IHME/GBD 2023) [17,18]. Where necessary, NFZ-derived patient counts were used to extrapolate the aggregated patient-reported estimates to the national population of patients treated for breast cancer in 2024.

The patient-reported components incorporated in the extended estimate originate from a survey conducted for the cited systemic report [12]. Self-reported data were collected from 289 women with breast-cancer recruited between June and September 2025. The final sample was determined by the number of complete questionnaires returned during the recruitment period rather than by probability sampling, and thus constitutes a pragmatic non-probability (convenience) sample; the questionnaire was not formally validated, and presenteeism and caregiver-related variables were captured as patient-reported proxy measures because no administrative source records them. Absenteeism and presenteeism were designed to capture conceptually distinct phenomena, namely time not worked versus reduced effectiveness during hours actually worked; each respondent could select only one response per questionnaire item, and administrative sickness-absence data (ZUS) and survey-derived components were assigned to separate categories. The cost category, cost bearer, population base, and time basis of each component, and the basis on which they are delineated from one another, are set out in Supplementary Materials (Table S7). We note, however, that selecting a single response per questionnaire item does not by itself demonstrate that the same lost time is not represented in more than one component. Residual overlap between reduced working time, presenteeism, unpaid work, and informal caregiving cannot be excluded from patient-reported data of this kind, and it is addressed among the limitations. Because this design does not guarantee statistical representativeness of the national breast cancer population, the corresponding cost components were confined to the extended estimate—presented as an exploratory scenario and subjected to probabilistic sensitivity analysis—rather than included in the core estimate, which relies exclusively on administrative and registry data.

As reported in [12], the survey was an anonymous online questionnaire open to any person with a breast-cancer diagnosis; all respondents were women, with a mean age of 47 years. Among respondents, 44% reported early-stage disease (0–II), 46% advanced disease (III–IV) and 10% could not specify the stage, while 24% reported treatment within a drug programme and 43% could not identify the organisational form of their treatment. Relative to the national population treated for breast cancer, the sample was younger (mean age 47 years, versus a national mean age at breast-cancer diagnosis of about 62 years (median 64; KRN, 2023)) and over-represented patients treated within a drug programme (24% versus 8.0% of NFZ-treated patients in 2024); the patient-reported components are therefore not nationally representative and are used only within the exploratory extended scenario. A comparison of the sample with national reference data is given in Supplementary Materials (Table S8). Individual responses were not re-analysed and the questionnaire is not reproduced; the values used here are the aggregated totals published in [12].

### 2.3. Disease Burden (Disability-Adjusted Life Years)

The disease burden of breast cancer was quantified using disability-adjusted life years (DALYs), following the Global Burden of Disease (GBD) framework [19,20]. One DALY represents the loss of one year of full health, combining years of life lost due to premature mortality (YLLs) and years lived with disability (YLDs):

DALY = YLLs + YLDs

Years of life lost (YLLs)

YLLs were calculated by multiplying the number of female breast-cancer deaths (ICD-10 C50) in each five-year age group (NCR, 2023) by the standard remaining life expectancy at the corresponding age of death, taken from the GBD 2019 Reference Life Table [21]. The age of death within each interval was approximated by the interval midpoint, and life expectancy obtained by linear interpolation; for the open-ended 85+ group a mean age of death of 90 years was assumed. In line with the GBD standard, base-case YLLs were undiscounted and not age-weighted; a variant discounting life-years at 3% was tested in sensitivity analysis. This health-related discount rate is conceptually distinct from, and applied independently of, the 5% rate used to discount monetary productivity losses; the two measure different quantities and are not conflated.

Years lived with disability (YLDs)

YLDs for female breast cancer in Poland were obtained directly from GBD 2023 (12,863; 95% UI 9007–17,861) [18].

Hybrid construction of the primary DALY estimate. The primary DALY figure combines registry-based YLLs with modelled GBD YLDs, and the two components do not originate from a single estimation framework. GBD mortality is adjusted for incomplete registration and for the redistribution of ill-defined causes of death, and GBD definitions and population coverage are harmonised across countries, whereas National Cancer Registry counts are unadjusted national records. In our assessment, the National Cancer Registry provides the best available evidence on breast-cancer mortality in Poland: it is the statutory national cancer registry and provides a complete enumeration of deaths rather than a modelled estimate, in a country with well-developed death-certification and cancer-registration systems. We therefore adopt registry-based YLLs as the primary mortality base, a choice reinforced by the design of this study: the same registered deaths enter the premature-mortality cost component, so that the epidemiological and the economic layers rest on a single, transparent national mortality base. We refer to this registry-anchored figure as the national-data estimate. The internally consistent native GBD 2023 estimate is reported alongside it as an internationally comparable alternative, not as a validation of the hybrid figure. GBD applies internationally calibrated adjustments—for registration incompleteness and cause-of-death redistribution—that are valuable for cross-country comparability but impose model-based assumptions on a setting with high-quality mortality data; the difference between the two estimates is confined to YLLs, and the applicability of these adjustments to the Polish setting is uncertain.

Relationship to the economic mortality estimate. The YLLs calculation and the premature-mortality productivity loss (Section 2.6) draw on the same NCR mortality source but, by design, on different death subsets: YLLs include all breast-cancer deaths across the full age range, whereas the productivity loss is restricted to deaths at working age. The two figures are therefore not expected to coincide; their difference reflects distinct constructs (years of healthy life lost versus economic output lost).

### 2.4. Cost Categories and Attribution to Cost Bearers

Costs were classified as direct medical, direct non-medical, and indirect, and attributed to four cost bearers: the healthcare payer (NFZ), the social-insurance system (ZUS), patients/households, and employers/the wider economy. Each cost component was mapped to its bearer to support the societal-perspective interpretation and the distributional analysis.

### 2.5. Direct Costs

#### 2.5.1. Public Healthcare Expenditures (NFZ)

Direct medical costs financed by the NFZ were obtained for breast-cancer-attributed services for 2024 across specialist outpatient care, inpatient care (including surgery), chemotherapy, radiotherapy, drug programmes, rehabilitation, and palliative/hospice care, based on NFZ settlement data for services attributed to ICD-10 C50 (and Z51 with concurrent C50). Primary care (POZ) is capitation-financed and not separately attributable.

These NFZ figures are a complete national enumeration of reimbursed breast-cancer care rather than a sample: they were obtained from the NFZ on request under the public-information procedure and cover all contracted providers nationwide, so no extrapolation was applied. Patients were identified by a primary diagnosis of C50, or a primary diagnosis of Z51 with a coexisting C50. In 2024, 231,422 patients were treated; this figure counts each patient once across service types, whereas the per-service counts may include the same patient under more than one service type and therefore overlap. Reimbursement was valued as the settled amount of each service where this exceeded zero or, for services reported within the hospital-network (PSZ) lump sum, at the PSZ reporting-unit price for the settlement period; emergency-department services were excluded. The data reflect NFZ records as of 28 April 2025.

#### 2.5.2. Patient Out-of-Pocket and Direct Non-Medical Costs

Patient-borne out-of-pocket medical expenditures and visit-related non-medical costs (transportation and other visit-related expenses) were obtained from the aggregated figures of the published systemic report [12] and extrapolated to the treated population (mean 17.0 visits/year). These components are reported within the extended estimate only.

### 2.6. Indirect Costs

Indirect costs were valued using a human-capital approach and included short-term work impairment (sickness absence and reduced working time), presenteeism, long-term productivity losses (premature mortality and disability), and losses related to informal caregiving and unpaid work. To avoid overstating losses when using average output per worker, a correction factor (β = 0.65) was applied, reflecting the marginal-to-average productivity ratio used in the European Commission 2024 Ageing Report projections [22] and consistent with AOTMiT HTA Guidelines 3.0 [23] and with the Polish methodological reports on the measurement of indirect costs [24,25]. This value is a methodological convention rather than a parameter estimated empirically for the Polish economy; it is applied to the six productivity components that are valued from GDP per employed person, namely premature mortality, disability, sickness absence, reduced working time, presenteeism, and informal caregiving, and it is therefore varied in both the deterministic and the probabilistic sensitivity analysis. It is not applied to unpaid work, which is valued at a replacement-cost rate for domestic help rather than from GDP per employed person; a marginal-to-average productivity adjustment has no meaning for a replacement-cost valuation, so that component is held constant when β is varied. Productivity was valued as GDP per employed person (GDP_2024_/P_2024_ × β), expressed per day (684 PLN, ≈€160.15) on the basis of 228 working days per year, and per hour (≈€20.02) on the assumption of an eight-hour working day, with future losses discounted at 5% annually [23]. All costs were calculated in PLN and converted to EUR at the NBP reference rate of 4.2730 PLN for 1 EUR (0.2340 EUR for 1 PLN) [26]. The human-capital approach was selected in preference to the friction-cost method because it is the method recommended for valuing indirect costs by the established Polish methodological guidance in this area [24,25], ensuring methodological comparability with other economic evaluations conducted in the country.

Employment concept and retirement (consistency). Employment rates applied to the premature-mortality and disability components were derived from the same national-economy employment concept used in the GDP-per-worker denominator (employed women by age from GUS national-economy statistics, divided by female population by age from GUS), ensuring internal consistency between the numerator (employment probability) and the denominator (GDP per employed person). A retirement-age threshold of 60 years for women was applied uniformly across both components.

Premature-mortality losses were estimated as, for each five-year age group, the number of deaths × discounted future production to retirement × age-specific employment rate × unit productivity. Disability losses were estimated as YLDs × unit productivity × employment rate, restricted to working ages. This is a modelling assumption rather than a direct measurement, and we present it as such: YLDs incorporate health-state disability weights and quantify time lived in reduced health, not years of employment or of productive capacity lost. The product should therefore be read as an approximation of the output foregone by women of working age living with breast cancer and its sequelae, not as an observed quantity. An empirically grounded alternative, based on disability-benefit receipt or on observed labour-force participation among patients, would be preferable; it would require individual-level social insurance data that were not available for the present analysis, and it is proposed as a direction for further work.

### 2.7. Uncertainty and Sensitivity Analysis

Given uncertainty in several parameters—particularly those underlying the extended estimate—we performed deterministic and probabilistic sensitivity analyses. One-way analyses varied key parameters individually (correction factor, discount rate, age-specific employment rates, apportionment of YLDs across the 50–69 band, and patient-reported means) and were summarised in a tornado diagram. Scenario analyses contrasted the core versus extended estimates and, for the burden layer, undiscounted versus 3%-discounted YLLs. Probabilistic sensitivity analysis used Monte Carlo simulation with distributions assigned to uncertain parameters (bootstrap-informed for patient-reported means), reporting 95% model-based simulation intervals for the core and extended societal costs. These intervals propagate the parameter uncertainty specified in the model; they are not sampling intervals derived from empirical distributions, and they do not capture structural uncertainty in the choice of components, the valuation method, or the extrapolation approach. All calculations of years of life lost, sensitivity analyses, and figures were produced in R (version 4.6.0; R Foundation for Statistical Computing, Vienna, Austria), using the stats package for Monte Carlo simulation and the ggplot2 package for graphics. The R code implementing the deterministic and probabilistic sensitivity analyses and the figures was written with AI assistance (Claude, Anthropic, v. 5.5); all analyses were executed and verified by the authors. In the one-way analysis, administrative inputs (NFZ, sickness absence) were varied by ±5–10%, the recomputed long-term components (premature mortality, disability) by ±15%, and the patient-reported components by ±30–35% around their 2024 base values. In the probabilistic analysis, each cost component was assigned a gamma distribution parameterised by its base value and an assumed coefficient of variation of approximately 3–5% (administrative), 10–12% (recomputed long-term), and 25–30% (patient-reported); components were treated as independent, as no empirical correlation structure was available. The deterministic ranges and coefficients of variation are analyst-assigned assumptions reflecting the presumed reliability of each data source rather than estimates derived from observed distributions; the width of the resulting interval around the core estimate consequently follows from those assumptions and should not be read as independent evidence of robustness. Independence is likewise a simplification: several patient-reported components are plausibly positively correlated, since a respondent reporting a large reduction in working time is also more likely to report high presenteeism and extensive caregiving needs. Ignoring such correlation narrows the simulated interval, so the reported interval for the extended scenario is more likely to understate than to overstate the true dispersion.

To further explore the model’s uncertainty three analyses were added. First, the correction factor β was varied over 0.55–0.80 (triangular distribution, mode 0.65) in both the one-way and the probabilistic analysis, scaling the six GDP-valued productivity components identified in Section 2.6 and leaving replacement-cost unpaid work unchanged. Second, four structural scenarios were examined: exclusion of unpaid work; exclusion of presenteeism; exclusion of the disability component from the core estimate; and valuation of premature mortality by the friction-cost approach instead of the human-capital approach, using a Poland-specific friction period of 38 days taken from published estimates of friction periods for OECD member countries [27]. Third, to test the independence assumption, the probabilistic analysis was repeated with positive correlation among the patient-reported components imposed through a Gaussian copula (rank correlation ρ = 0.3 and ρ = 0.6), with β held at 0.65 so that the effect of correlation is isolated. The simulation intervals reported below therefore rest on different assumptions and are not nested. The baseline interval treats the components as independent and holds β at 0.65; the β interval adds uncertainty in β while retaining independence; and the correlation intervals add rank correlation among the patient-reported components while holding β at 0.65. The two scenarios were run separately and were not combined, so no reported interval reflects both sources of uncertainty at once.

## 3. Results

### 3.1. Disease Burden (DALYs)

**Figure 1:**
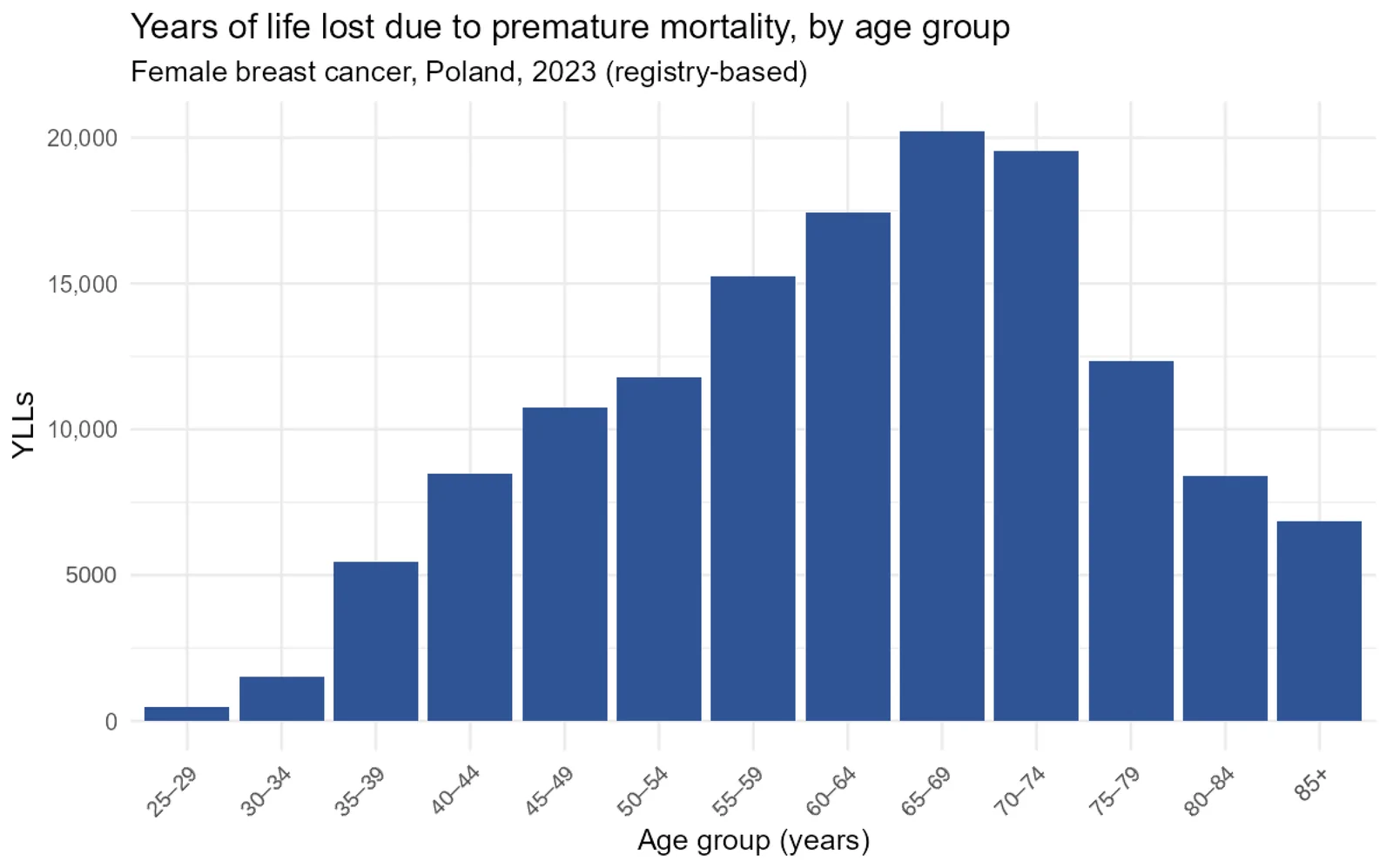
Years of life lost due to premature mortality (YLLs) attributable to female breast cancer in Poland, 2023, by age group (registry-based).

In 2023, 6757 women died of breast cancer in Poland. These deaths corresponded to 138,492 YLLs. Adding 12,863 YLDs (GBD 2023), the total burden of female breast cancer was estimated at 151,355 DALYs. Propagating only the uncertainty in the YLD component (95% UI 9007–17,861) while holding the registry-based YLL as a fixed point estimate yields a partial 95% interval of 147,499–156,353 DALYs; this interval is incomplete, as it excludes uncertainty in registry-based mortality and therefore understates the true dispersion. Premature mortality accounted for 91.5% of the burden, indicating that breast cancer in Poland is driven far more by fatal than by non-fatal outcomes; on average, each death entailed a loss of 20.5 years of life. The burden of premature mortality was concentrated in the 60–74 age range (Figure 1). The full age-group breakdown of YLLs, YLDs, and DALYs, together with crude and directly age-standardised rates per 100,000 women, is given in Supplementary Materials (Table S1). No uncertainty interval is reported for the registry-based YLL, because the National Cancer Registry publishes death counts without an accompanying measure of estimation uncertainty. The hybrid DALY total therefore has no complete interval and should not be read as being as precise as the native GBD 2023 estimate, for which an interval is available. The unquantified part of its uncertainty concerns the completeness of death registration and cause-of-death coding, and is of the order suggested by the difference between the two estimates reported in Table 1.

### 3.2. NFZ-Financed Direct Medical Costs (2024)

Breast-cancer care financed by the NFZ across outpatient, inpatient (including surgery), chemotherapy, radiotherapy, drug-programme, rehabilitation, and palliative settings amounted to €837,690,279 in 2024 (Table 2). Drug-program pharmacotherapy represented the largest share.

### 3.3. Direct Costs Borne by Patients (2024)

Total direct costs borne by patients—out-of-pocket medical expenditures plus visit-related non-medical costs—were estimated at €322,418,254 (Table 3). (Extended tier.)

### 3.4. Indirect Costs

#### 3.4.1. Short-Term Absenteeism

Breast-cancer-related sick-leave days exceeded 1.4 million in 2024, yielding absenteeism costs of over €230 million (core tier; absence volumes from ZUS, the production loss borne by employers and the economy). Reduced working time, derived from patient-reported data, added €574,023,110 (extended tier) (Table 4 and Table 5).

#### 3.4.2. Long-Term Productivity Losses

Long-term losses comprised premature mortality and disability, estimated on a consistent national-economy employment concept with mortality from NCR 2023 and productivity valued at 2024 levels. Premature mortality amounted to €196.6 million and disability to €145.7 million, for a long-term total of €342,338,576 (Table 6). (Core tier.)

Disability sensitivity: because GBD provides YLDs in broad age bands, the 50–69 band was apportioned between working (50–59) and post-retirement ages in proportion to the female population; the plausible range was €128.7–145.8 million.

#### 3.4.3. Presenteeism

Presenteeism losses were estimated at €679,752,053 (extended tier) (Table 7).

#### 3.4.4. Informal Caregiving

Caregiver productivity losses totalled €565,462,755 (extended tier) (Table 7).

#### 3.4.5. Unpaid Work

Losses in unpaid work, valued by replacement cost, reached €982,710,695 (extended tier) (Table 7).

### 3.5. Total Economic Burden and Cost Structure

**Figure 2:**
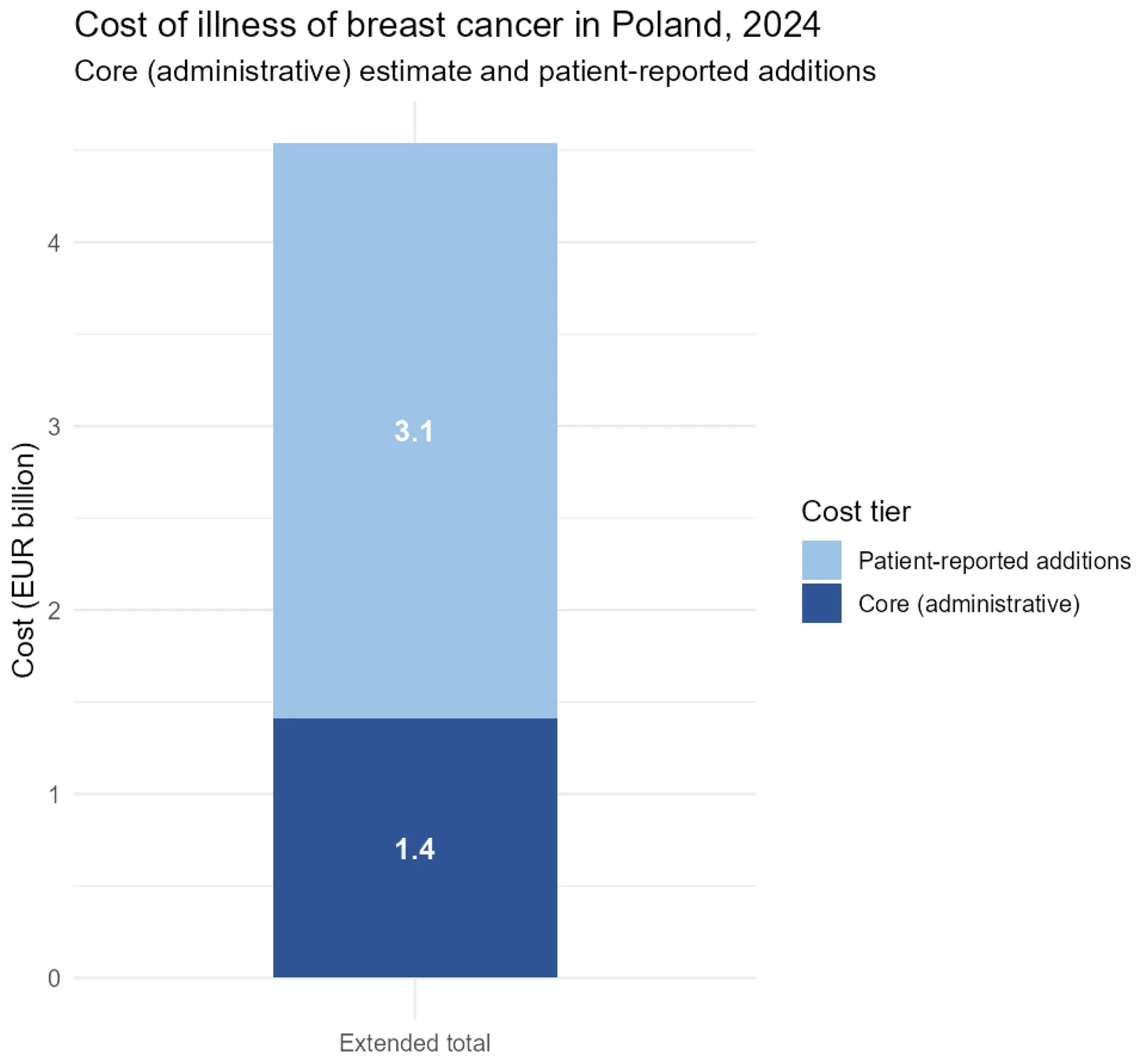
Cost of illness of breast cancer in Poland, 2024: core (administrative) estimate and patient-reported additions that together form the exploratory extended total (EUR billion).

**Figure 3:**
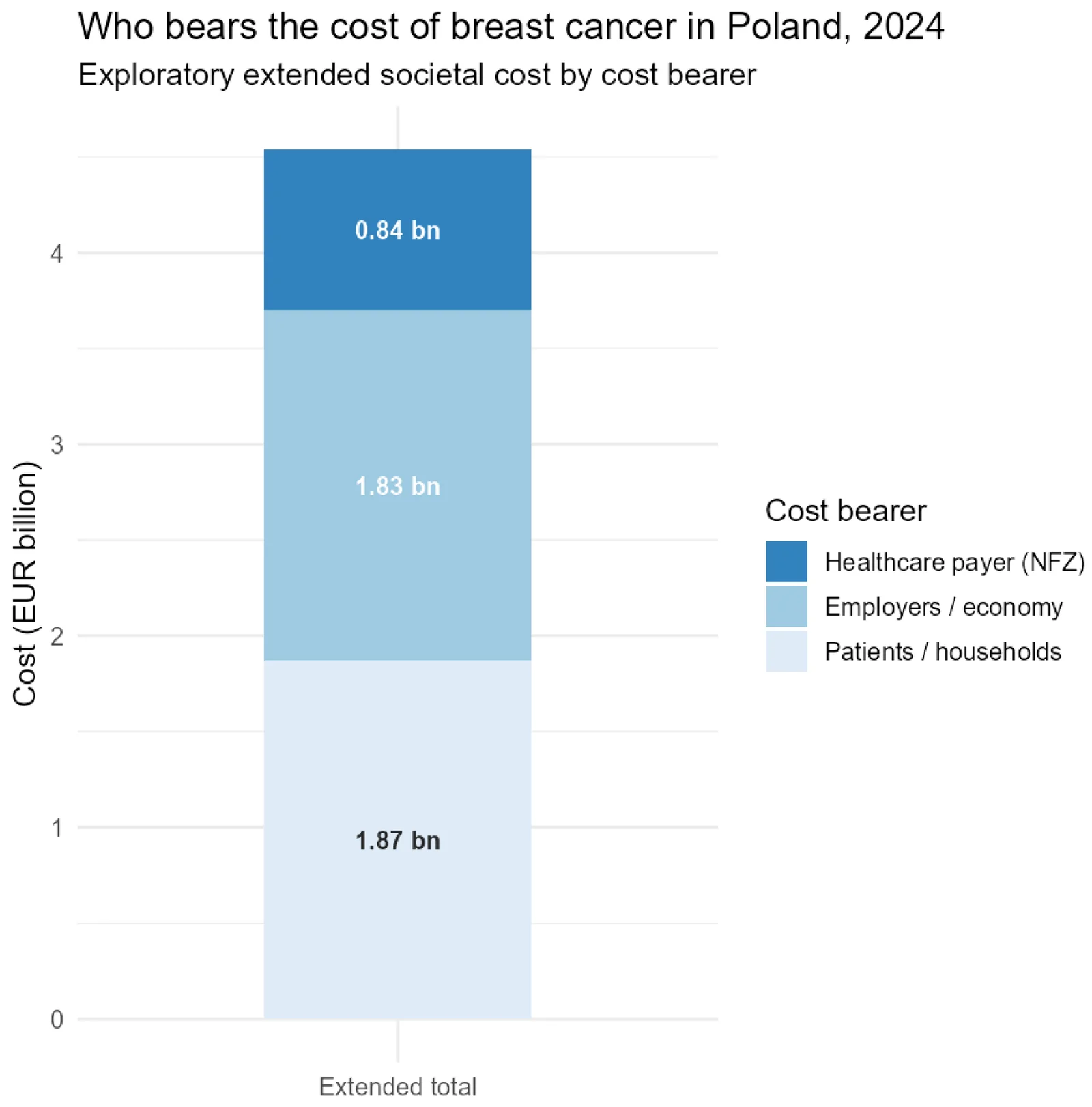
Attribution of the exploratory extended societal cost of breast cancer in Poland, 2024, to cost bearers (EUR billion).

The core societal cost of breast cancer—based exclusively on administrative, registry, and public-statistical sources (NFZ, ZUS, KRN, and GUS)—was approximately €1.41 billion. Adding the patient-reported components yielded an exploratory extended scenario of approximately €4.54 billion (Figure 2). Indirect costs represented approximately three-quarters of the extended total; among them, unpaid work, presenteeism, reduced working time, and informal caregiving were the leading components. In the core estimate, by contrast, direct medical expenditure (NFZ, €837.7 million) exceeded total indirect costs (€574.9 million), so that indirect costs became dominant only once patient-reported components were added. A substantial share of the burden arose outside the healthcare system: attribution to cost bearers showed that the public payer (NFZ) accounted for 18.5% of the extended total, patients/households bore 41.2%, and employers/the economy 40.3% (Table 7; Figure 3). Production losses from sickness absence, although quantified using ZUS records, are borne by employers and the wider economy rather than by the social-insurance system.

### 3.6. Sensitivity Analysis

**Figure 4:**
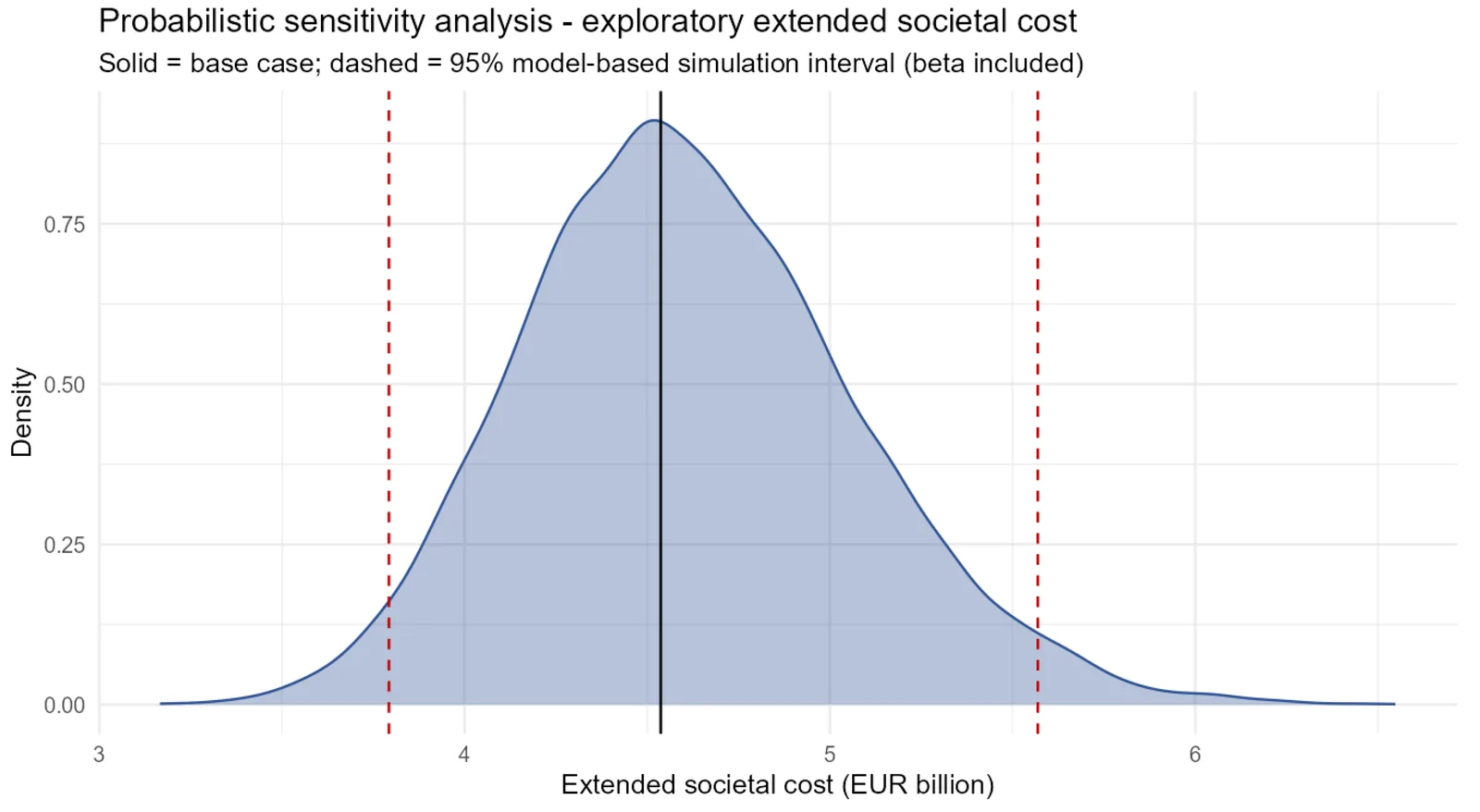
Probabilistic sensitivity analysis of the exploratory extended societal cost (Monte Carlo, 10,000 iterations); solid line, base case; dashed lines, the 95% model-based simulation interval from the run that includes uncertainty in β (€3.79–5.57 billion). The baseline interval reported in Section 3.6 (€3.81–5.40 billion) holds β at 0.65.

**Figure 5:**
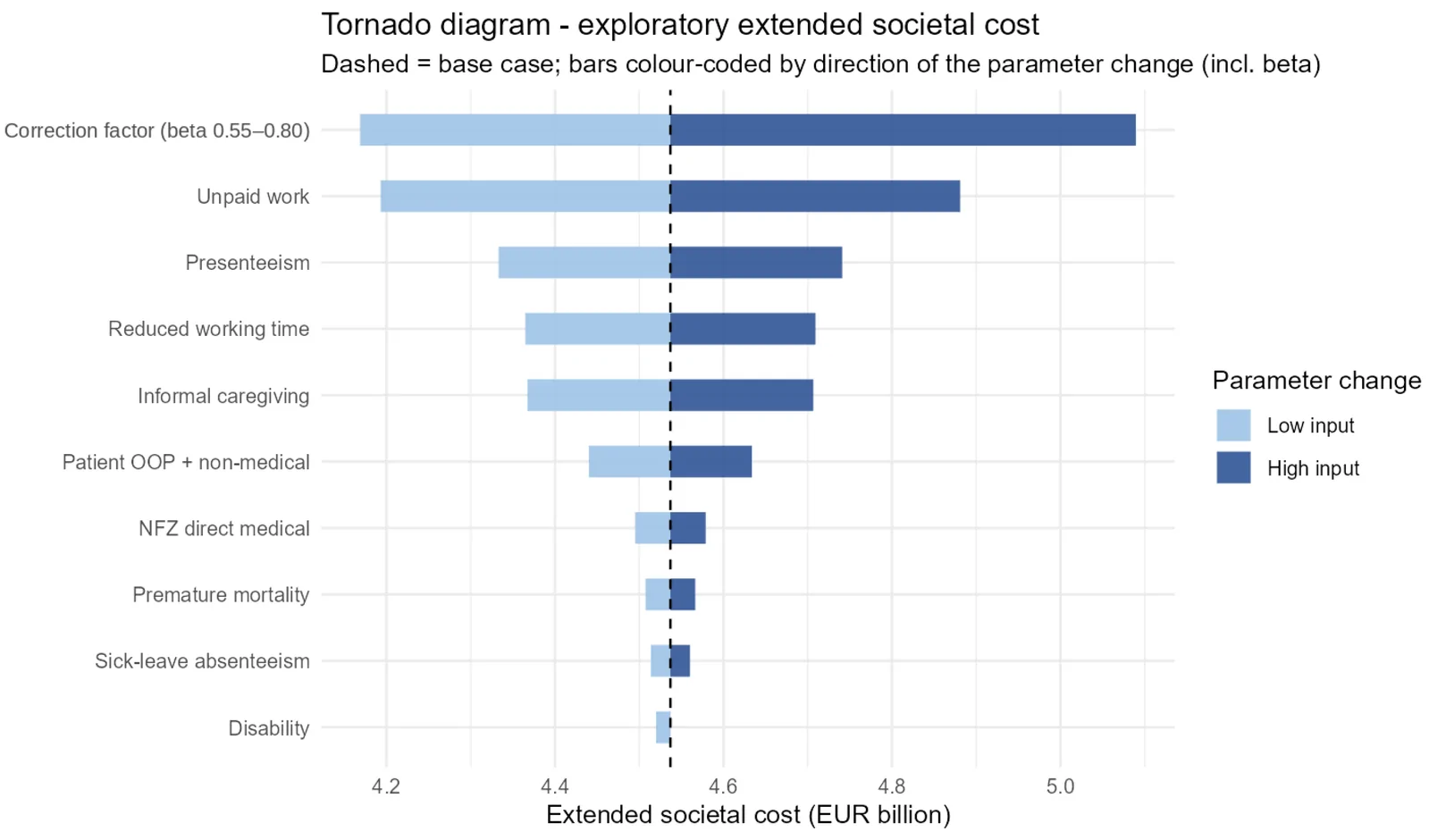
Tornado diagram of one-way sensitivity of the exploratory extended societal cost to the individual cost components and to the correction factor β; bars are colour-coded by the direction of the parameter change.

The sensitivity analysis used the 2024 base-case component values reported in Table 7, with the one-way ranges and probability distributions specified in Section 2.7; all cost results refer to 2024. Probabilistic sensitivity analysis confirmed that the two tiers differ markedly in robustness. The simulated interval around the core societal cost was narrow (mean €1.41 billion; 95% model-based simulation interval €1.34–1.49 billion). This width follows directly from the low coefficients of variation of approximately 3% to 5% assigned to the administrative components, and it is therefore a property of those assumptions rather than independent evidence that the estimate is robust. The exploratory extended scenario was substantially more dispersed (mean €4.54 billion; 95% model-based simulation interval €3.81–5.40 billion) (Figure 4). One-way sensitivity analysis showed that this uncertainty was driven almost entirely by the patient-reported components—unpaid work, presenteeism, reduced working time, and informal caregiving—whereas the administrative and recomputed long-term components contributed little to the overall variation (Figure 5). These results indicate that the core estimate rests on the more reliable inputs, while the extended figure remains exploratory. Neither interval captures structural uncertainty, and neither should be read as an upper bound on the national cost.

When the correction factor β was varied (triangular, 0.55–0.80), it became the single most influential parameter in the one-way analysis (swing €0.92 billion across the range); including β in the probabilistic analysis widened the simulation intervals to €1.32–1.55 billion (core) and €3.79–5.57 billion (extended). In the structural scenarios, excluding unpaid work reduced the extended estimate to €3.55 billion and excluding presenteeism to €3.86 billion; excluding the disability component reduced the core estimate to €1.27 billion; and valuing premature mortality by the friction-cost approach (Poland-specific friction period of 38 days [27]) reduced the core estimate to €1.22 billion and the extended estimate to €4.35 billion. Finally, allowing positive correlation among the patient-reported components widened the extended interval from €3.81–5.40 billion under independence to €3.52–5.74 billion (ρ = 0.3) and €3.33–6.02 billion (ρ = 0.6), confirming that the assumption of independence understates the dispersion of the extended estimate. Because the β scenario and the correlation scenarios were run separately, neither of these intervals combines the two sources of uncertainty; an interval reflecting both would be wider than any reported here.

## 4. Discussion

Breast cancer constitutes a substantial and growing economic burden in Poland, driven by both direct healthcare expenditures and indirect costs related to productivity loss. Our findings are consistent with previous Polish evidence documenting a steady rise in total costs and a large indirect component [7,11], and with earlier work identifying long-term effects such as disability and premature mortality as major drivers of productivity losses [8].

These estimates should be read against a rising national trend. Between 2013 and 2023 the number of new breast-cancer cases among Polish women increased from 17,481 to 21,716 (a 24% rise; age-standardised incidence 89.8 to 103.8 per 100,000, European standard), while deaths increased from 5816 to 6757 [16]. The continued rise in incidence, only partly offset by broadly stable age-standardised mortality, points to a growing future burden and reinforces the policy relevance of the present estimates.

The dual burden–cost framing adds a further dimension. The disease burden of 151,355 DALYs was dominated by years of life lost, which accounted for 91.5% of the total. Because the YLD component is derived from a modelled source and is reported in broad age bands, we draw no stronger inference about the relative importance of fatal and non-fatal outcomes than this decomposition supports. For external comparison, the GBD 2023 study estimated a higher burden for female breast cancer in Poland (174,567 YLLs, 12,863 YLDs, and 187,430 DALYs; 95% UI 175,009–199,775). The difference in YLLs reflects GBD’s modelled adjustment of registry data for under-registration and garbage-code redistribution, which typically yields higher cancer-mortality estimates than raw national registries. Our registry-based figure is therefore likely to be the lower of the two and is internally consistent with the mortality used in the cost analysis, while the GBD 2023 estimate is internationally comparable and internally consistent across its YLL and YLD components.

International evidence further supports these observations. A systematic review of triple-negative breast cancer demonstrated that both direct and indirect costs increase substantially with disease progression and treatment intensity, with wide variability across healthcare systems [28]. Similarly, a cost-of-illness study from Turkey showed that indirect costs constitute the dominant component of the economic burden, exceeding direct medical expenditures and accounting for the majority of total costs [29]. Consistent findings are reported in a recent systematic review by Franklin et al. [30], which highlighted that indirect costs—particularly those related to productivity loss and informal caregiving—are substantially underrepresented in the literature; when included, they may exceed direct healthcare costs, underscoring the importance of adopting a societal perspective. The review also emphasised considerable heterogeneity across studies and concluded that current evidence captures only part of the total economic burden of breast cancer, limiting its usefulness for policy-making [30].

Reporting results in two tiers is intended to address this heterogeneity transparently. Many indirect categories cannot be measured from routine administrative data and rely on patient-reported estimates, introducing methodological uncertainty. By separating a core estimate grounded entirely in administrative and registry data (≈€1.41 billion) from an extended estimate incorporating patient-reported components (≈€4.54 billion), we make explicit which portion of the burden rests on the more reliable inputs and which is an exploratory scenario whose direction of bias cannot be determined from a non-probability sample. Even the core estimate substantially exceeds public-payer expenditure alone, reinforcing that analyses limited to healthcare budgets understate the societal impact.

The structure of indirect costs is also important for interpretation. Evidence from Poland indicates that productivity losses are driven not only by short-term absenteeism but also by long-term effects such as disability and premature mortality [8]. Broader approaches incorporating presenteeism and caregiver-related productivity losses provide a more comprehensive estimate of the societal burden, and these components are increasingly recognised as essential elements of cost-of-illness analyses [30]. The structure of direct costs observed here is consistent with national and international evidence: in Poland, pharmacological treatment—particularly within drug programmes—accounts for the largest share of direct expenditures, followed by surgery and radiotherapy [11], a pattern also reflected internationally, where systemic therapies are consistently identified as the primary cost driver [6]. The economic burden also varies substantially by disease stage, with advanced and metastatic disease associated with markedly higher expenditures [6,28,29], highlighting the economic importance of early detection and timely treatment.

A comment on the treatment actually financed is warranted, together with a clear statement of what these data can and cannot show. NFZ settlement records document which services were financed and at what cost; they contain no information on disease stage, regimen, guideline concordance, or clinical outcome, so the present analysis cannot assess whether the treatment administered to any individual patient was clinically appropriate. What the composition of expenditure does describe is the shape of the care that was delivered. Active antineoplastic treatment dominates, with drug programmes, chemotherapy, and radiotherapy together accounting for 58.4% of NFZ expenditure and drug programmes alone for 35.6%, a share consistent with the reimbursement of innovative systemic agents, although an expenditure share does not by itself establish how many patients received them. Care remains strongly hospital-centred: inpatient services absorbed 32.7% of expenditure against 3.9% for specialist outpatient care, a ratio of roughly eight to one that reflects the organisation and financing of oncology services rather than clinical need alone. The most striking feature is the marginal position of rehabilitation, at 0.7% of direct medical expenditure (€6.2 million), set against €145.7 million of productivity loss attributed to disability in the same population, a difference of more than twentyfold. Palliative and hospice care accounted for 4.3%. These proportions say nothing about the appropriateness of individual treatment decisions, but they do indicate that the financing of breast-cancer care in Poland is concentrated on active treatment, while the survivorship and functional-recovery services that plausibly bear on the disability burden receive a very small share. Establishing whether treatment is appropriate in the clinical sense would require patient-level data linking stage, regimen, and outcome to guideline recommendations; such data were not available here, and we propose this as a direction for further work.

Several factors help to explain how these results relate to international estimates and how they may evolve. Cross-country differences in the reported cost of breast cancer reflect not only price and wage levels but also the structure of treatment pathways and reimbursement: the large share of direct costs attributable to drug programmes in Poland is sensitive to the reimbursement of innovative systemic therapies, so that decisions to reimburse new agents can materially shift both the level and the composition of direct expenditure. We further hypothesise that the cost structure will change as organised mammographic screening matures, since earlier detection shifts the stage distribution towards locoregional disease, which is associated with lower treatment intensity [6,28,29]. The present analysis does not test this: it compares neither screened with unscreened women nor costs across disease stages, and it contains no model of screening, so no conclusion about the economic effect of screening follows from our results. Finally, the divergence between our registry-based DALY estimate and the higher GBD 2023 figure is itself informative: it indicates the order of magnitude by which modelled and registry-based mortality estimates can differ. Whether registry counts understate mortality in Poland was not tested here, and this remains a hypothesis rather than a finding of the present study.

A methodological strength of this analysis is the internal consistency of its productivity valuation. Employment probabilities and the GDP-per-worker unit were derived from a single national-economy employment concept, and a uniform retirement-age threshold was applied across the mortality and disability components, avoiding the mixing of employment concepts that can inflate long-term estimates. This yields long-term productivity losses (€342.3 million) that are internally coherent with the disease-burden layer through their shared registry mortality base.

The findings have direct implications for health technology assessment (HTA) in Poland. The currently binding Polish HTA guidelines (AOTMiT 3.0) take the public-payer perspective as the reference case, with indirect costs reported only as supplementary information. Notably, the draft update to these guidelines, released for public consultation in 2026 [31], foresees a more prominent role for the societal perspective and for the human-capital valuation of indirect costs—an approach already recommended by established Polish methodological guidance in this area [24,25]. Against this evolving background, the magnitude of the productivity losses documented here indicates that evaluations restricted to the public-payer perspective may systematically understate the value of interventions that preserve employment and prevent premature death. The component estimates reported here are intended as inputs to such analyses. A cost-of-illness study cannot establish the cost-effectiveness of any intervention, and we therefore make no claim about the value of particular therapies or of screening; whether the inclusion of indirect components changes a reimbursement decision can be determined only within a full economic evaluation. More broadly, the results argue for routine reporting of a societal-perspective scenario alongside the payer perspective in Polish oncology HTA submissions, and for methodological consistency in how employment and productivity are valued, so that indirect costs are neither omitted nor inflated. Given the dominant role of drug-programme expenditure in direct costs, pricing and reimbursement policy for innovative therapies remains a central determinant of the overall economic burden.

This study has several limitations. The components included in the extended estimate were derived from a survey of 289 women with breast cancer conducted for the cited systemic report. The absence of random sampling and the limited representativeness of this sample are important limitations, particularly for extrapolation to the entire population of women treated for breast cancer in Poland. At the same time, given the burdensome nature of cancer and the sensitivity of the topics addressed—financial situation, limitations of labour-market activity, unpaid work, and informal caregiving—obtaining complete data from 289 patients is a valuable result, providing information on cost categories that are not recorded in routine administrative databases and therefore cannot be estimated from NFZ, ZUS, or public-registry data alone. Accordingly, the core estimate, based primarily on administrative, registry, and statistical data, constitutes the principal and more reliable result of the analysis, whereas the extended estimate should be regarded as a supplementary, exploratory scenario indicating the potential scale of additional costs borne by patients, households, and the economy; its results should not serve as a standalone basis for precise quantitative conclusions, but rather to highlight cost categories omitted from analyses restricted to the public-payer perspective. The analysis relied on data from different years: costs and productivity for 2024, mortality for 2023, and patient-reported monetary values carrying the price level of the 2024 to 2025 survey recall period. This reflects data availability at the time of analysis, and the resulting mismatch between reference periods remains a genuine source of uncertainty in the aggregate estimate; the extended estimate in particular is not a constant-price figure. The disability component is additionally constrained by the broad age bands in which YLDs are reported, addressed through sensitivity analysis. Finally, the analysis was restricted to female breast cancer; male cases (approximately 1% of the total) were not modelled, which has a negligible effect on the aggregate estimates. Several further limitations should be stated. The questionnaire underlying the extended components was not formally validated, and residual overlap between reduced working time, presenteeism, unpaid work, and informal caregiving cannot be excluded, so those components may in part capture the same lost time twice. Extrapolating per-patient means to the national treated population assumes that the surveyed women resemble that population in age, stage, and employment status, which cannot be verified for a non-probability sample. Disability-related productivity loss is derived from YLDs, which measure time lived in reduced health rather than employment actually lost. In the baseline probabilistic analysis the cost components were simulated as independent, which narrows the reported interval. The correlated scenarios reported in Section 3.6 quantify that effect, and those intervals, rather than the baseline one, give the better indication of the dispersion of the extended estimate. Primary care expenditure is omitted because capitation financing cannot be attributed to a diagnosis. Costs falling outside the health and labour spheres are not captured at all, including psychological consequences, quality-of-life losses, health effects on caregivers, and long-term household financial consequences; the estimates therefore describe a defined set of measurable components rather than the full social and economic consequences of the disease. Social welfare payments stand on a different footing: they are excluded by design rather than omitted. As set out in Section 1, sickness and disability benefits are transfers that redistribute income rather than societal resource costs, and adding them to the societal total would double count the productivity loss they compensate. Finally, PLN values were converted at the year-end National Bank of Poland reference rate, so the EUR figures are sensitive to the choice of conversion date. Re-expressing all costs at the 2024 annual-average NBP reference rate (4.3065 PLN/EUR, the mean of the daily Table A rates [32]) rather than the year-end rate (4.2730 PLN/EUR [26]) changed the core estimate from €1.41 to €1.40 billion and the extended estimate from €4.54 to €4.50 billion (a reduction of about 0.8%), confirming that the choice of conversion date is not material. Differences in methodology across studies further limit direct comparability [30].

## 5. Conclusions

Female breast cancer imposes a substantial, multidimensional burden in Poland that extends far beyond the healthcare system. Expressed epidemiologically, the disease caused 151,355 DALYs in 2023, overwhelmingly due to premature mortality. Expressed economically, its core societal cost—grounded entirely in administrative and registry data—was approximately €1.41 billion. The exploratory extended scenario, which additionally incorporates patient-reported components extrapolated from a non-probability sample of 289 women, reached approximately €4.54 billion; it is a model-based scenario rather than a national estimate. Direct medical expenditure was the single largest component of the core estimate, whereas indirect costs dominate the extended scenario.

The distribution of costs across stakeholders shows that, in addition to the public healthcare payer, a considerable share of the burden is borne by patients, households, and the broader economy through lost productivity, underscoring the importance of a comprehensive societal perspective. From a policy standpoint, the size of the productivity component indicates that evaluations restricted to the payer perspective omit a large part of the loss. Whether investment in early detection, treatment, or survivorship care is economically worthwhile cannot be answered by a cost-of-illness study and requires a full economic evaluation, for which the component estimates reported here may serve as inputs. A more holistic understanding of the economic burden of breast cancer is essential for designing policies that improve clinical outcomes while mitigating broader social and economic consequences. Taken together, the findings indicate that public healthcare expenditure represented approximately 18.5% of the modelled extended estimate, so that analyses limited to healthcare budgets address only part of the measurable burden of breast cancer in Poland.

## Figures and Tables

**Table 1:** Disease burden of female breast cancer in Poland, 2023 (DALYs).

Measure	Value	95% UI
Years of life lost (YLLs), registry-based	138,492	—
Years lived with disability (YLDs), GBD 2023	12,863	9007–17,861
DALYs (primary, hybrid)	151,355	—
DALYs, GBD 2023 native (cross-check)	187,430	175,009–199,775

**Table 2:** NFZ-financed direct medical costs of breast cancer in Poland, 2024 (EUR). Note: the table covers expenditure financed by the National Health Fund only. Costs borne directly by patients, comprising out-of-pocket medical purchases and visit-related non-medical costs, do not appear in NFZ settlement data and are reported separately in Table 3 within the exploratory extended scenario. Primary care is capitation-financed and cannot be attributed to a diagnosis. Values are presented in EUR to allow international comparison; all calculations were performed in PLN and converted at the National Bank of Poland reference rate for 31 December 2024 (4.2730 PLN for 1 EUR) [26].

Scope of Care	Cost (EUR)	Share
Specialist outpatient care (AOS)	32,347,558	3.9%
Drug programme	297,842,505	35.6%
Chemotherapy	23,588,463	2.8%
Radiotherapy	167,528,304	20.0%
Inpatient care (excl. drug programme, chemotherapy, radiotherapy)	274,093,412	32.7%
Rehabilitation	6,150,103	0.7%
Palliative and hospice care	36,139,935	4.3%
Total	837,690,279	100.0%

**Table 3:** Total direct costs borne by patients, 2024 (EUR). Component-level sources, denominators, unit values, and formulas are provided in Supplementary Materials (Table S2).

Component	Cost (EUR)
Visit-related non-medical costs	128,460,698
Out-of-pocket medical expenditures	193,957,556
Total	322,418,254

**Table 4:** Economic parameters used in the valuation of productivity losses, 2024. Component-level sources, denominators, unit values, and formulas are provided in Supplementary Materials (Table S3).

Parameter	Value
Gross domestic product, 2024 (EUR)	852,143,692,956
Employed persons, 2024	15,169,658
Correction factor (β)	0.65
Working days per year	228
Value of one lost working day (EUR)	160.15
Value of one lost working hour (EUR)	20.02

**Table 5:** Short-term productivity losses, 2024 (EUR). Note: sick-leave absenteeism is a core-tier component derived from ZUS records, whereas reduced working time is an extended-tier component derived from patient-reported data. The two are shown together for completeness, and the combined total should not be interpreted as a single estimate of uniform reliability. Component-level sources, denominators, unit values, and formulas are provided in Supplementary Materials (Table S4).

Component	Cost (EUR)
Sick-leave absenteeism (1,452,329 days), core tier	232,590,489
Reduced working time, extended tier	574,023,110
Total short-term (mixed tiers)	806,613,599

**Table 6:** Long-term productivity losses (recomputed), 2024 (EUR). Component-level sources, denominators, unit values, and formulas are provided in Supplementary Materials (Table S5).

Component	Cost (EUR)	Share
Premature mortality	196,627,078	57.4%
Disability	145,711,498	42.6%
Total long-term	342,338,576	100.0%

**Table 7:** Economic burden of breast cancer in Poland, 2024, by component, tier, and cost bearer (EUR). Component-level sources, denominators, unit values, and formulas are provided in Supplementary Materials (Table S6).

Component	Tier	Cost Bearer	Cost (EUR)
NFZ direct medical	Core	Healthcare payer (NFZ)	837,690,279
Sick-leave absenteeism	Core	Employers/economy	232,590,489
Premature mortality	Core	Employers/economy	196,627,078
Disability	Core	Employers/economy	145,711,498
Core subtotal			1,412,619,344
Patient out-of-pocket + non-medical	Extended	Patients/households	322,418,254
Reduced working time	Extended	Employers/economy	574,023,110
Presenteeism	Extended	Employers/economy	679,752,053
Informal caregiving	Extended	Patients/households	565,462,755
Unpaid work	Extended	Patients/households	982,710,695
Extended total			4,536,986,211

Attribution by bearer (extended total): healthcare payer (NFZ) €837.7 million (18.5%); employers/economy €1828.7 million (40.3%); patients/households €1870.6 million (41.2%). Production losses from sickness absence, recorded via ZUS, are attributed to employers and the wider economy rather than to the social-insurance system.

## Data Availability

The administrative, registry, and statistical inputs are publicly available from the sources cited. Component-level data sources, unit values, calculation formulas, and the detailed valuation methods are set out in the Supplementary Materials. The derived analytical dataset, the full list of input parameters and unit values, and the R code used for the DALY calculation and for the deterministic and probabilistic sensitivity analyses, together with the figures, are provided as Supplementary Materials. Together, the Supplementary Materials permit full reproduction of the core estimate. The patient-reported components of the extended estimate were not re-analysed for this study; they are reproduced from the aggregated values published in the source report [12]. Individual-level survey data and the study questionnaire are not made available. Further requests may be addressed to the corresponding author.

## Supplementary Materials

The following supporting information can be downloaded at https://www.mdpi.com/article/10.3390/healthcare14203434/s1. Table S1: Disease burden of female breast cancer in Poland by age group, 2023; Table S2: Direct costs borne by patients, 2024, sources and calculation; Table S3: Economic parameters used in the valuation of productivity losses, 2024, sources and calculation; Table S4: Short-term productivity losses, 2024, sources and calculation; Table S5: Long-term productivity losses, 2024, sources and calculation; Table S6: Economic burden by component, tier, and cost bearer, 2024, sources and calculation; Table S7: Basis of each cost component and its delineation from the others; Table S8: Characteristics of the patient-reported survey sample compared with national reference data; File S1: input parameter workbook; File S2: R code for the DALY calculation, the sensitivity analyses, and the figures.

## Abbreviations

The following abbreviations are used in this manuscript:

AOTMiT	Agency for Health Technology Assessment and Tariff System
CF	correction factor
DALY	disability-adjusted life year
GBD	Global Burden of Disease
GDP	gross domestic product
GUS	Statistics Poland (Główny Urząd Statystyczny)
HTA	health technology assessment
ICD-10	International Classification of Diseases, 10th Revision
IHME	Institute for Health Metrics and Evaluation
NCR	National Cancer Registry
NFZ	National Health Fund (Narodowy Fundusz Zdrowia)
OOP	out-of-pocket
PSA	probabilistic sensitivity analysis
UI	uncertainty interval
YLD	years lived with disability
YLL	years of life lost
ZUS	Social Insurance Institution (Zakład Ubezpieczeń Społecznych)
