# Supplementary material for: Disease Burden and Cost of Illness of Female Breast Cancer in Poland: A Societal-Perspective Analysis with Cost Attribution to Payers, Patients, and the Economy
Source: Healthcare (Basel). 2026 Oct 14;14(20):3434. doi: 10.3390/healthcare14203434 (PMC13704402; doi:10.3390/healthcare14203434)
Supplement: Supplementary file 1 [file healthcare-14-03434-s001.zip › Supplementary Tables post Rev R1.pdf]

Supplementary materials

This appendix provides the age-group breakdown of the disease burden (Table S1), and the component-level data sources, population denominators, unit values, calculation formulas, and data years underlying Tables 3–7 of the main text, together with the detailed calculation methods set out at the end of this appendix. Core components (NFZ direct medical costs, sick-leave absenteeism, premature mortality, and disability) are fully reproducible from these specifications together with the Supplementary parameter sheet and R code. Extended, patient-reported components are cited as aggregated values from the source report [11] and are not re-derived here; fields that are, by design, not reproduced are marked "n/a — cited aggregate [11]."

Symbol key (2024 base): K_year = (GDP_{2024} / E_{2024}) × β = €36,514 (156,021 PLN); K_day = K_year / 228 = €160.15; K_hour = K_day / 8 = €20.02. GDP_{2024} = €852,143,692,956 (3,641,210,000,000 PLN @ 4.2730 PLN/EUR, NBP reference rate 31 Dec 2024); E_{2024} = 15,169,658 employed persons (GUS); β = 0.65; r = 5% (monetary discount); statutory female retirement age = 60. NFZ-treated breast-cancer population, 2024: n = 231,422.

Table S1. Disease burden of female breast cancer in Poland by age group, 2023: YLL, YLD, DALY, and crude and age-standardized rates per 100,000 women (cf. main Table 1).

Age group	YLL	YLD	DALY	Female population	DALY per 100,000 women	YLL per 100,000 women
25-29	493	51	544	1,047,539	51.9	47.1
30-34	1,532	189	1,721	1,286,977	133.7	119.0
35-39	5,437	436	5,873	1,504,448	390.4	361.4
40-44	8,483	707	9,190	1,560,317	589.0	543.7
45-49	10,748	1,017	11,765	1,443,372	815.1	744.6
50-54	11,772	1,094	12,866	1,209,841	1,063.5	973.0
55-59	15,232	1,179	16,411	1,123,313	1,461.0	1,356.0
60-64	17,431	1,724	19,155	1,264,193	1,515.2	1,378.8
65-69	20,230	2,120	22,350	1,383,868	1,615.0	1,461.8
70-74	19,528	1,821	21,349	1,201,795	1,776.4	1,624.9
75-79	12,347	1,187	13,534	763,072	1,773.6	1,618.1
80-84	8,420	715	9,135	520,885	1,753.7	1,616.5
85+	6,838	609	7,447	581,934	1,279.6	1,175.0
<25	0	14	14	4,592,351	0.3	0.0
**Total (crude)**	**138,491**	**12,863**	**151,354**	**19,483,905**	**776.8**	**710.8**
**Age-standardized (ESP 2013)**					**722.3**	**661.2**

YLL, years of life lost; YLD, years lived with disability; DALY, disability-adjusted life years; ESP 2013, 2013 European Standard Population. Sources: YLL — National Cancer Registry (KRN), female C50 deaths 2023, projected against the GBD 2023 reference life table; YLD — GBD 2023 (female, breast cancer, Poland); female population — Statistics Poland (GUS), balance as of 30 June 2023. DALY = YLL + YLD (registry–GBD hybrid). Crude rates are per 100,000 women in the corresponding age group; the age-standardized rate is directly standardized to the 2013 European Standard Population. Figures may not sum exactly owing to rounding. Age-group uncertainty intervals are available only for YLD (GBD) and are not propagated to DALY; no interval is reported for registry-based YLL. Burden below age 25 (YLD only) is shown as a single row.

Table S2. Direct costs borne by patients, 2024 (EUR) — sources and calculation (cf. main Table 3).

Component	Cost (EUR)	Tier	Data source	Population denominator	Unit value	Formula	Data year
Visit-related non-medical costs	128,460,698	Extended	[11], aggregated (patient-reported)	n/a — cited aggregate [11]	n/a — cited aggregate [11]	n/a — cited aggregate [11]	2024
Out-of-pocket medical expenditures	193,957,556	Extended	[11], aggregated (patient-reported)	n/a — cited aggregate [11]	n/a — cited aggregate [11]	n/a — cited aggregate [11]	2024

Total	322,418,254	Extended	[11], aggregated	—	—	Sum of components above	2024

All rows are extended (patient-reported) and are cited from [11] rather than re-derived.

Table S3. Economic parameters used in the valuation of productivity losses, 2024 — sources (cf. main Table 4).

Parameter	Value	Unit	Data source	Data year	Definition / formula
Gross domestic product (GDP_{2024})	852,143,692,956	EUR	GUS, nominal GDP 2024 (3,641,210,000,000 PLN @ 4.2730)	2024	National accounts; converted at NBP reference rate 31 Dec 2024
Employed persons (E_{2024})	15,169,658	persons	GUS, national-economy employment, Dec 2024	2024	National-economy employment concept (consistent numerator/denominator)
Correction factor (β)	0.65	—	EC AWG 2012 / D'Auria 2010; AOTMiT HTA 3.0	—	Marginal-to-average productivity adjustment
Discount rate (monetary, r)	5%	per year	AOTMiT HTA 3.0	—	Applied to long-horizon (premature-mortality) losses
Working days per year	228	days	Assumption, net of statutory leave	2024	8 working hours per day
Value of one lost working day (K_day)	160.15	EUR	Derived	2024	K_day = (GDP/E) × β × (1/228)
Value of one lost working hour (K_hour)	20.02	EUR	Derived	2024	K_hour = K_day / 8

Table S4. Short-term productivity losses, 2024 (EUR) — sources and calculation (cf. main Table 5).

Component	Cost (EUR)	Tier	Data source	Population denominator	Unit value	Formula	Data year
Sick-leave absenteeism	232,590,489	Core	ZUS, administrative (sick-leave days, ICD-10 C50)	ZUS-recorded C50 sick-leave days (national)	K_day = €160.15	$Days_{ee}$ (ZUS, C50) × K_day	2024
Reduced working time	574,023,110	Extended	[11], aggregated (patient-reported)	n/a — cited aggregate [11]	n/a — cited aggregate [11]	n/a — cited aggregate [11]	2024
Total short-term	806,613,599	—	Mixed (core + extended)	—	—	Sum of components above	2024

Absenteeism is administrative (ZUS) and core; reduced working time is patient-reported and cited from [11]. The two were designed to capture different quantities (time not worked versus a reduction in paid working time reported by the patient), but because the latter is self-reported, a residual overlap with recorded sick leave cannot be excluded.

Table S5. Long-term productivity losses (recomputed), 2024 (EUR) — sources and calculation (cf. main Table 6).

Component	Cost (EUR)	Share	Tier	Data source	Population denominator	Unit value	Formula	Data year
Premature mortality	196,627,078	57.4%	Core	Recomputed: KRN 2023 deaths × 2024 productivity	Female C50 deaths 2023 by age (working-age subset; total 6,757)	K_year = €36,514	Eq. (E3): $\Sigma_a D_a \times \Sigma_t[(GDP/E)\times\beta\times ER_{a+t}\times(1+r)^{-}{}_{t}]$, t to age 60	Deaths 2023; values 2024
Disability	145,711,498	42.6%	Core	Recomputed: GBD 2023 YLD × 2024 productivity	YLD by age (GBD 2023)	K_year = €36,514	Eq. (E4): $\Sigma_a YLD_a \times [(GDP/E)\times\beta\times ER_a]$ (undiscounted)	YLD 2023; values 2024
Total long-term	342,338,576	100.0%	—	Recomputed	—	—	Sum of components above	2024

Health outcomes (deaths, YLD) are dated 2023 (latest available); monetary values are at 2024 base. Disability uses undiscounted YLD; the YLD-allocation range (€128.7–145.8 million) is carried into the sensitivity analysis. ER = employment rate by age group.

Table S6. Economic burden by component, 2024 (EUR) — sources and calculation (cf. main Table 7; tier and cost bearer are given there).

Component	Cost (EUR)	Tier	Data source	Population denominator	Unit value	Formula	Data year
NFZ direct medical	837,690,279	Core	NFZ, administrative (primary C50, or primary Z51 with coexisting C50)	NFZ-treated BC population 2024 (n = 231,422)	Reimbursed amounts	Sum of service-area reimbursements (AOS, drug programme, chemotherapy, radiotherapy, inpatient net, rehabilitation, palliative/hospice); POZ excluded	2024
Sick-leave absenteeism	232,590,489	Core	ZUS, administrative (C50 sick-leave days)	ZUS C50 sick-leave days (national)	K_day = €160.15	$Days_{ee}$ × K_day	2024
Premature mortality	196,627,078	Core	KRN 2023 deaths × 2024 productivity	C50 deaths 2023 by age	K_year = €36,514	Eq. (E3)	2023 / 2024
Disability	145,711,498	Core	GBD 2023 YLD × 2024 productivity	YLD by age (GBD 2023)	K_year = €36,514	Eq. (E4)	2023 / 2024
Core subtotal	1,412,619,344	—	—	—	—	Sum of core components	2024
Patient out-of-pocket + non-medical	322,418,254	Extended	[11], aggregated (patient-reported)	n/a — cited aggregate [11]	n/a — cited aggregate [11]	n/a — cited aggregate [11]	2024
Reduced working time	574,023,110	Extended	[11], aggregated (patient-reported)	n/a — cited aggregate [11]	n/a — cited aggregate [11]	n/a — cited aggregate [11]	2024
Presenteeism	679,752,053	Extended	[11], aggregated (patient-reported)	n/a — cited aggregate [11]	n/a — cited aggregate [11]	n/a — cited aggregate [11]	2024
Informal caregiving	565,462,755	Extended	[11], aggregated (patient-reported)	n/a — cited aggregate [11]	n/a — cited aggregate [11]	n/a — cited aggregate [11]	2024
Unpaid work	982,710,695	Extended	[11], aggregated (replacement cost)	n/a — cited aggregate [11]	n/a — cited aggregate [11]	n/a — cited aggregate [11]	2024
Extended total	4,536,986,211	—	—	—	—	Core subtotal + extended components	2024

Core components are fully reproducible from the Supplementary parameter sheet and R code. Extended components are cited from [11] as aggregated values and are not re-derived. The full NFZ extraction specification (service-selection rules, handling of patients with multiple services, C50/Z51 de-duplication, and extrapolation formulae) is provided separately in response to reviewer R3 (Methods C3).

Detailed calculation methods

Unit values. The value of one lost working day (K_day) and hour (K_hour) were derived from GDP per employed person, adjusted by the correction factor β:

$$(E1)\quad K\_day = (GDP_{2024} / E_{2024}) \times \beta \times (1 / WD_{2024}),\quad \text{with } WD_{2024} = 228$$

$$(E2)\quad K\_hour = K\_day / 8$$

Premature mortality (core). Losses were valued with a human-capital approach as the discounted stream of future production lost among women who died at working age, using age-specific deaths from the National Cancer Registry, age-specific employment rates, and GDP per employed person adjusted by β, projected to the statutory retirement age:

$$(E3)\quad C\_mort = \Sigma_a D_a \times \Sigma_{t=0}^{T_a} [(GDP/E) \times \beta \times ER_{a+t} \times (1 + r)^{-t}]$$

where D_a = deaths in age group a; ER_{a+t} = employment rate at age a + t; r = 0.05; T_a = remaining working years to age 60.

Disability (core). Losses were valued from years lived with disability (YLD) combined with age-specific employment rates and GDP per employed person adjusted by β (undiscounted):

$$(E4)\quad C\_dis = \Sigma_a YLD_a \times [(GDP/E) \times \beta \times ER_a]$$

Extended components (patient-reported). In the source report [11], reduced working time, presenteeism, informal caregiving, and unpaid work were valued from patient-reported time losses using the same GDP-based human-capital unit values (K_hour or K_day), except unpaid work, which was valued at a replacement-cost rate for domestic help (35.50 PLN per hour, 2025). The present study cites the resulting aggregated component totals and does not reproduce the underlying per-patient computations or the survey instrument; these components are reported only within the exploratory extended estimate.

Delineation of cost components and residual overlap

Table S7. Basis of each cost component and its delineation from the others.

Component	Tier	Cost category	Cost bearer	Population base	Quantity / time basis	Data source
NFZ direct medical	Core	Direct medical	Healthcare payer (NFZ)	NFZ-treated BC patients, 2024	Reimbursed services, calendar year 2024	NFZ
Sick-leave absenteeism	Core	Indirect – productivity	Employers / economy	Insured workers on C50 sick leave	Full working days not worked (recorded sick leave), 2024	ZUS
Premature mortality	Core	Indirect – productivity	Employers / economy	Female C50 deaths 2023	Future working years lost to death (fatal; YLL basis), discounted	KRN
Disability	Core	Indirect – productivity	Employers / economy	YLD 2023	Non-fatal disability time during life (YLD basis)	GBD
Patient out-of-pocket + non-medical	Extended	Direct, non-reimbursed	Patients / households	Patient-reported [11]	Out-of-pocket and non-medical spending	[11]
Reduced working time	Extended	Indirect – productivity	Employers / economy	Patient-reported [11]	Patient-reported reduction in paid working time (beyond recorded sick leave)	[11]
Presenteeism	Extended	Indirect – productivity	Employers / economy	Patient-reported [11]	Reduced effectiveness during hours actually worked	[11]
Informal caregiving	Extended	Indirect – time	Patients / households	Patient-reported [11]	Caregivers' time devoted to the patient	[11]
Unpaid work	Extended	Indirect – time	Patients / households	Patient-reported [11]	Patient's own forgone unpaid/domestic work (replacement cost)	[11]

The components were defined so as not to overlap, and the four axes set out below are the basis on which their totals are summed. This is a design argument rather than a demonstration: for the patient-reported components, whose delineation rests on self-report, a residual overlap cannot be excluded, and this is stated among the limitations of the main text. (i) The cost categories do not overlap: direct medical (payer), direct non-reimbursed (household), and indirect productivity/time costs are distinct. (ii) Fatal and non-fatal burden are separated — premature mortality captures future production lost to death (YLL basis), disability captures non-fatal loss (YLD basis). (iii) Within productivity, absenteeism measures full working days not worked, drawn from administrative ZUS sick-leave records; presenteeism measures reduced effectiveness during hours actually worked; and reduced working time measures the patient-reported reduction in paid working time. Administrative (ZUS) and patient-reported ([11]) sources are collected independently, and the patient-reported components were delineated in the source report [11] so as not to overlap with recorded sick leave. (iv) Informal caregiving (a caregiver's time) and unpaid work (the patient's own forgone unpaid activity) concern different persons' time.

Representativeness of the survey sample

Table S8. Characteristics of the patient-reported survey sample [11] compared with national reference data.

Characteristic	Survey sample (n = 289) [11]	National reference	Source
Sex	100% women	Breast cancer overwhelmingly affects women	[11]; KRN
Mean age	47 years	Mean age at diagnosis ≈ 62 years (median ≈ 64); only 21% of incident cases before age 50	[11]; KRN (2023 incidence by age)
Treated within a drug programme	24%	8.0% (18,552 of 231,422 NFZ-treated patients, 2024)	[11]; NFZ
Disease stage	Early (0–II) 44%; advanced (III–IV) 46%; unknown 10%	Not publicly available — KRN does not publish a national stage distribution	[11]
Economically inactive owing to illness	26% (of whom 66% had left work)	—	[11]
Sampling frame	Self-selected, non-probability online convenience sample	Complete national enumeration (NFZ administrative data)	[11]; NFZ

The survey sample was a self-selected online convenience sample and is not nationally representative: it skews younger and over-represents patients treated within a drug programme (and, correspondingly, advanced-stage disease) relative to the national population treated for breast cancer. For this reason the patient-reported components are confined to the exploratory extended estimate. The National Cancer Registry (KRN) does not publish a national stage distribution, so a stage-specific comparison is not possible from public data. The survey sample (mean age 47 years) is about 15 years younger than the national mean age at breast-cancer diagnosis (≈ 62 years; median ≈ 64), and only 21% of national incident cases arise before age 50 (KRN, 2023 incidence by age). Stage cannot be compared because KRN does not publish a national stage distribution.
